# Whispers of Pathogens; Social Contagion in Infectious Disease Dynamics: A Review

**DOI:** 10.1002/hsr2.72463

**Published:** 2026-04-28

**Authors:** Sayed Mortaza Fayez

**Affiliations:** ^1^ Student Research Committee, School of Medicine Mashhad University of Medical Sciences Mashhad Iran

**Keywords:** digital epidemiology, health equity, infectious disease dynamics, social contagion, social determinants of health, syndemic framework

## Abstract

**Background and Aims:**

Infectious disease dynamics are deeply intertwined with social structures, behaviors, and information systems. This narrative review examines how social factors conceptualized through the integrated lens of “social contagion” within a syndemic framework shape infectious disease patterns from January 2000 to January 2024.

**Methods:**

Literature was retrieved from PubMed, Scopus, Web of Science, and Google Scholar using targeted combinations of terms related to social determinants, networks, communication, and digital epidemiology. Foundational pre‐2000 works were consulted for theoretical grounding. Studies were selected based on their relevance to understanding how social processes influence infectious disease dynamics. The selection process prioritized high‐impact empirical studies, meta‐analyses, and seminal theoretical works that collectively informed the four thematic areas presented. As this is a narrative review, no statistical analyses were performed. The synthesis approach followed established guidelines for narrative reviews.

**Results:**

Four major themes emerged from the synthesis of identified literature: (1) structural and commercial determinants socioeconomic inequality, racism, and commercial practices produce environments where infectious and non‐communicable diseases interact synergistically; (2) network effects household, occupational, and mobility patterns shape transmission pathways and superspreading events; (3) belief systems misinformation, behavioral contagion, and varying levels of institutional trust influence vaccine uptake and protective behaviors; and (4) diagnosis and intervention digital epidemiology and agent‐based models offer tools for integrating social data into disease surveillance and response.

**Conclusion:**

Infectious diseases are fundamentally biosocial phenomena. Effective control requires moving beyond biomedical models toward approaches centered on social epidemiology, equity, and trust‐building. Future research must employ transdisciplinary collaboration to better measure and model the complex interactions that constitute social contagion.

## 1 Introduction

Infectious diseases have long been framed as biological encounters between a host and a pathogenic agent. The progression of an epidemic depends on two factors: its microbiological aspects and its human societal structures that shape its progress [1, 2, 3, 4]. The 21st century has provided stark demonstrations of this reality: the HIV/AIDS pandemic illuminated the role of stigma and inequality in shaping geographic patterns of transmission [5]; the 2009 H1N1 influenza pandemic revealed how global air travel networks enable rapid pathogen spread; the West African Ebola virus disease outbreak (2014–2016) and subsequent outbreaks in the Democratic Republic of the Congo exposed the deadly interplay between institutional mistrust, cultural practices, and fragile health systems; and the COVID‐19 pandemic offered an unprecedented case study in how socioeconomic status, occupation, household density, information ecosystems, and policy responses collectively determine individual and population‐level risk [6, 7].

The narrative review demonstrates that infectious diseases exist as social and biological realities according to research from source [8]. Social factors operate as intelligence networks which interact with each other to create different patterns of disease spread and danger levels, which researchers use to evaluate and control diseases [9]. The review uses social contagion as its main concept to study how social networks transmit various elements, including ideas and behaviors, which results in changes to disease transmission patterns [2, 10]. The title's “whispers” metaphorically represent the subtle, often overlooked social channels through which disease risk is communicated, amplified, or mitigated, ranging from stigma accompanying diagnosis to the viral spread of protective (or harmful) information through digital networks.

This review offers an integrative synthesis that combines biosocial, syndemic, and social network frameworks to examine how social contagion processes operate across multiple levels of analysis. Rather than claiming a theoretically novel framework, the contribution lies in synthesizing existing perspectives to reveal their interconnections. The review achieves three objectives through its research work, which includes. The first objective of this study requires the creation of a framework which unites three different levels of analysis, while the second objective focuses on studying how these levels generate disease patterns that affect entire populations. The researchers used this integrated framework to study social contagion between the years 2000 and 2024, which led to digital technologies causing major changes in social contagion patterns. The study demonstrates how information spreads throughout networks, which creates feedback loops that affect current methods of disease transmission.

The objective of this review is to synthesize evidence from January 2000 to January 2024, elucidating the multifaceted impact of social factors on infectious disease dynamics. We will examine the structural determinants that create landscapes of vulnerability, the network topologies that define transmission pathways, the cognitive and informational landscapes that shape behavior, and the novel tools and frameworks needed to understand and intervene in this interconnected system.

## 2 Methodology

The research design of this study uses a narrative review because it effectively combines different research studies from various fields of study, that include epidemiology, sociology, network science, and digital health to create a unified framework. The research method of narrative reviews enables researchers to study complex multidimensional phenomena while developing unified theoretical frameworks, which systematic reviews cannot achieve through their standard assessment procedures [11, 12]. The review adheres to established guidelines, which describe the recommended practices for narrative reviews according to existing standards [12].

### 2.1 Search Strategy

The researchers conducted systematic searches in PubMed, Scopus, Web of Science, and Google Scholar databases to identify relevant literature which existed between January 2000 and January 2024. The researchers performed search queries which utilized terms from five different thematic domains: (1) structural determinants: (“social determinants” OR “socioeconomic factors” OR “structural determinants”) AND (“infectious disease” OR “epidemic” OR “pandemic”); (2) network effects: (“social network” OR “contact network”) AND (“disease transmission”); (3) information ecosystems: (“health communication” OR “misinformation” OR “vaccine hesitancy”) AND (“infectious disease”); (4) digital epidemiology: (“digital epidemiology” OR “social media”) AND (“surveillance”); and (5) stigma: (“stigma” OR “discrimination”) AND (“infectious disease”). Foundational theoretical works published before 2000 were included to provide historical and conceptual context.

### 2.2 Study Selection and Inclusion Criteria

The studies met the inclusion criteria because they studied infectious disease dynamics as they related to social factors, and because the studies were published in peer‐reviewed journals or as authoritative books/book chapters, and because the content was written in English, and because the research provided empirical data and methodological innovations and theoretical contributions which helped to understand social contagion processes. The final synthesized literature showed that researchers from high‐income countries conducted most of the research, although the study did not impose any geographic limitations which were addressed throughout the discussion section. Studies were excluded because they studied biomedical aspects without exploring social factors or because they researched non‐infectious conditions without showing how they affected infectious disease dynamics.

### 2.3 Screening and Selection Process

The first search produced around 1850 distinct records after the removal of duplicate entries. The research team assessed titles and abstracts to determine their connection to the study's goals. The researchers conducted full‐text evaluations on 320 studies, which showed potential relevance. The synthesis process started with 51 selected sources, which researchers combined with 8 essential pre‐2000 sources. The selection process used these criteria to choose studies which included: (a) high‐impact empirical studies with rigorous methodologies; (b) systematic reviews and meta‐analyses which provided evidence that had been synthesized; (c) essential theoretical works which helped develop the discipline; and (d) studies which demonstrated research across multiple geographic locations and various population groups.

Theme identification and synthesis: The four major themes presented in the results were identified through an iterative process of thematic analysis. The initial coding process began after the research team became acquainted with all the literature, which had been chosen for study. The researchers created candidate themes from these codes, which they later examined and clarified through discussions with professionals who understood the subject matter. The final thematic structure (1) structural and commercial determinants, (2) network effects, (3) belief systems, and (4) diagnosis and intervention represents a synthesis of the literature that captures the multi‐level nature of social contagion processes while remaining grounded in the evidence base.

### 2.4 Statistical Analysis

As this is a narrative review synthesizing qualitative and quantitative literature, no statistical tests were performed. No a priori levels of significance were defined, and no statistical software packages were used for analysis. The synthesis approach was descriptive and interpretive, following established methods for narrative reviews.

### 2.5 Limitations of the Methodology

As a narrative review, this synthesis is inherently selective rather than exhaustive. The lack of official quality evaluation tools leads researchers to discuss study limitations through qualitative methods instead of conducting systematic assessments. The wide interdisciplinary approach requires researchers to give up detailed analysis of specific disciplines so they can create new theoretical frameworks. The review shows evidence from digital contagion research, which has undergone fast changes because it represents a current scientific field that continues to evolve.

## 3 Results

The results of the literature revealed four interconnected thematic areas through which social contagion processes shape infectious disease dynamics. Before presenting these themes, it is important to note that the evidence base is characterized by several tensions and gaps. First, while extensive research documents associations between social factors and disease outcomes, causal mechanisms remain incompletely understood, particularly regarding how macro‐level structural factors translate into individual‐level infection risk. Second, methodological approaches vary widely, with network studies often capturing fine‐grained interaction data but limited geographic scope, while ecological studies achieve broad population coverage but lack mechanistic detail. Third, the literature disproportionately represents high‐income countries, raising questions about the generalizability of findings to low‐ and middle‐income settings where social structures and disease burdens differ substantially. These limitations qualify the confidence with which conclusions can be drawn and highlight priorities for future research.

### 3.1 Structural and Commercial Determinants: Creating Landscapes of Vulnerability

Disease incidence is not randomly distributed; it maps systematically onto preexisting social fault lines. Structural determinants of the unequal distribution of power, resources, and wealth fundamentally shape population‐level vulnerability to infectious diseases [13]. Socioeconomic status (SES) emerges consistently as the most powerful predictor of infectious disease incidence. Tuberculosis (TB), HIV, hepatitis, and respiratory infections disproportionately affect lower SES populations through multiple pathways: overcrowded housing facilitates airborne and contact transmission; occupational exposures concentrate risk among essential workers with high contact rates; nutritional inadequacies compromise immune function; and barriers to healthcare access delay diagnosis and treatment [14, 15]. The COVID‐19 pandemic provided particularly stark evidence of these mechanisms, with hospitalization and mortality rates 2–3 times higher in disadvantaged communities across wealthy nations [16].

Critical to interpreting this evidence, commercial determinants of health, defined as private sector activities affecting health outcomes, increasingly shape infectious disease vulnerability [17]. The aggressive marketing of ultra‐processed foods has contributed to global increases in obesity and diabetes, which lead to severe respiratory infection outcomes that include influenza and COVID‐19 [18]. Industrial agricultural practices create zoonotic diseases through their two main pathways, which include environmental destruction and methods of raising animals in large quantities [19]. The practice of using antibiotics on food animals creates a situation which leads to increasing antimicrobial resistance because it makes bacterial infections harder to treat [20]. The business practices function through market systems, which enable them to make profits while putting public health at risk because they construct frameworks which result in higher infectious disease transmission rates.

Healthcare access becomes more difficult for people because of two factors, which include structural racism along with gender inequality. Historical and contemporary discrimination produce residential segregation, concentrated poverty, and differential exposure to environmental hazards [21]. For HIV, gender‐based violence, unequal power dynamics in sexual relationships, and stigma surrounding female sex work create pathways of vulnerability that prolong transmission [22]. The different social forces which exist today create a situation where syndemic conditions develop through simultaneous disease outbreaks, which become more severe because of the negative social circumstances that exist in the community. The presence of HIV, TB, and substance use disorders has reached higher levels among people who come from marginalized communities, yet these three diseases show a pattern of existing together [23].

#### 3.1.1 Critical Synthesis

While the association between structural factors and infectious disease outcomes is well‐established, important methodological limitations warrant consideration. The majority of research studies use ecological designs, which fail to establish individual‐level causation, while residual confounding continues to be a problem. Research from low‐income settings remains scarce because it limits understanding of structural determinants that operate in extreme poverty, weak governance, and high infectious disease environments. The existing research uses structural factors to establish their relationship with infection risk because direct observation of these mechanisms remains unavailable. The upcoming research work needs to focus on three specific research methods, which include longitudinal studies and natural experiments for policy evaluation and mixed‐methods research that studies how people experience their vulnerable structural situations.

### 3.2 Network Effects: Contact Structures, Community Dynamics, and Mobility Patterns

Pathogens travel along the pathways of human interaction. Social network analysis has fundamentally transformed understanding of transmission dynamics by shifting focus from population aggregates to the structure of connections through which pathogens spread [24]. Contact networks exhibit characteristic structures: households, schools, workplaces, healthcare facilities, and recreational venues each create distinct patterns of interaction that shape pathogen movement [25]. Empirical studies using diary‐based contact surveys have quantified age‐specific mixing patterns, revealing that children drive transmission of respiratory infections through school‐based contacts, while adults bridge transmission between households and communities [25].

The phenomenon of superspreading observed in SARS, MERS, Ebola, and COVID‐19 illustrates the critical role of network heterogeneity. Superspreading events occur when an individual with high connectivity or high infectiousness transmits pathogens to a disproportionately large number of secondary cases [26]. Network analysis reveals that superspreading is not random but concentrated in particular settings, such as religious gatherings, nightclubs, and crowded workplaces, where contact patterns enable explosive transmission. Understanding these network structures enables targeted interventions, such as closing specific venue types rather than implementing blanket restrictions.

The international spread of new pathogens emerges through worldwide population movements, which predominantly utilize air travel systems. Network models demonstrate that H1N1(2009) and SARSCoV2(2020) spatial distribution depended on worldwide airline network connectivity as the most important factor [27]. The transmission patterns within countries depend on three types of local mobility: commuting flows, neighborhood movement, and retail visits [28, 29]. The COVID‐19 mobile phone location data established that different socioeconomic groups experienced various mobility reduction levels, which directly affected their infection rates because lower‐income people who lacked remote work capabilities faced constant infection risk [30].

Social networks provide protective benefits to people who use them. High social capital communities trust their members and practice reciprocity, while their residents engage in civic activities, which results in more effective public health information sharing and better mutual aid during quarantines and increased compliance with group recommendations [31]. The network structures of marginalized communities function as a double‐edged sword because their dense interconnected networks enable fast information flow and social backing, but also lead to fast pathogen spread when many people face health risks at once [32].

#### 3.2.1 Critical Synthesis

Network epidemiology has advanced understanding of transmission mechanisms, yet important gaps remain. Most network studies rely on cross‐sectional contact data that cannot capture dynamic changes in interaction patterns during epidemics. Data from low‐income countries remains sparse, despite different social structures (multigenerational households, dense informal settlements) that may fundamentally alter transmission dynamics. Methodological challenges in measuring sexual and drug‐use networks limit understanding of blood‐borne pathogen transmission. Furthermore, the protective effects of social capital have been inconsistently measured and may operate differently across cultural contexts.

### 3.3 Belief Systems: Information Ecosystems, Misinformation, and Behavioral Contagion

Human behavior, mediated by perceptions and beliefs, represents the ultimate determinant of transmission dynamics. The Health Belief Model, together with its related theories, explains that people will adopt protective behaviors through vaccination and mask usage and social distancing only when they observe their personal vulnerability to the disease and its effects, and they understand the advantages of protective measures and the challenges that make it hard to adopt those measures [33]. Social interaction creates the conditions that lead people to develop these particular perceptions. “Behavioral contagion” describes the way health behaviors spread through social networks as people who share social ties make joint decisions about vaccination and mask usage [34].

Critically, digital transformation has changed all information environments, which now serve as the basis for establishing and sharing health beliefs. Social media platforms enable public health messages to reach a wide audience, while they also create conditions for the quick spread of both false and misleading information [35]. The World Health Organization classified an “infodemic” which described COVID‐19 as having excessive information that included both true and false content during the pandemic [36]. Vaccine misinformation spreads through social networks, which show characteristics of biological transmission as vaccine‐related myths about the debunked MMR‐autism connection and COVID‐19 vaccine rumors circulate through network‐based systems and grow through accounts that function as superspreaders [37].

Trust in institutions, government, science, and healthcare systems constitutes a critical social resource during infectious disease outbreaks. Historical medical abuses (Tuskegee syphilis study, unethical research in colonized populations) and contemporary discrimination have produced profound medical mistrust in marginalized communities [8]. During the West African Ebola outbreak, mistrust of foreign healthcare workers contributed to delayed treatment‐seeking and community resistance to control measures [38]. During COVID‐19, lower vaccine uptake in racial and ethnic minority communities reflected not simply misinformation exposure but rational responses to structural discrimination and historical betrayal [38, 39].

#### 3.3.1 Critical Synthesis

Research on health beliefs and misinformation faces significant methodological challenges. Cross‐sectional surveys assess attitudes at specific times, yet they fail to prove how information exposure affects behavioral changes. The experimental studies that investigate how misinformation correction works show varied results because some studies show that fact‐checking can accidentally strengthen incorrect beliefs through backfire effects. The measurement of trust remains contested because different studies operationalize the concept through different operational definitions, which restrict their ability to compare results. The examination of personal beliefs leads to an incomplete understanding of how people behave because vaccine uptake needs both public acceptance and affordable vaccine services to be successful.

### 3.4 Diagnosis and Intervention: Surveillance, Modeling, and Digital Epidemiology

Recognizing the centrality of social factors demands new approaches to surveillance and modeling. Traditional surveillance systems monitor pathogen distribution; social surveillance must additionally track the spread of risk factors, vulnerability conditions, and behavioral patterns. Digital epidemiology leverages real‐time data from online searches, social media, mobility applications, and wearable devices to provide insights into disease activity and population responses [40, 41]. Google Flu Trends pioneered this approach by correlating influenza‐like illness search queries with surveillance data, while Twitter analysis has enabled real‐time monitoring of vaccination sentiment and misinformation spread [42].

A critical examination reveals that epidemiological models have developed methods to include social complexity in their mathematical approaches. The research used agent‐based models (ABMs), which create virtual environments for mixed groups of people who interact through changing social networks to study how social factors such as stigma‐related testing refusal and knowledge distribution and policy‐related behavior changes affect the success of medical programs [43, 44]. The ABMs of COVID‐19 showed that socioeconomic status, job‐related risks, and home environment differences between people made apparently neutral policies worsen existing inequalities [30]. The use of social determinant information in models enhances predictability and supports the development of fair policies according to research [45].

The social aspects of disease control must be addressed through methods that operate at multiple levels. The process of reducing stigma provides essential support for controlling infectious diseases [46]. HIV, TB, and Ebola‐related stigma prevent people from getting tested and following treatment and disclosing their status, which leads to further transmission of the disease. The program needs three important elements to succeed; first, educational programs should reach the community; second, affected people should take part in program creation; and finally, the program needs to implement structural modifications that will decrease discrimination [47]. Trust‐based communication methods should be developed specifically for different community groups. The method of using community leaders whom people trust, along with cultural figures and healthcare workers, has more success than using direct communication methods from organizations. Digital platforms show potential for “prebunking” which protects users from deceptive content through controlled exposure to fake arguments that will calm their minds about the actual information [48].

Structural interventions address root causes. Pandemic preparedness cannot be reduced to biomedical countermeasures; it requires policies addressing socioeconomic inequality, paid sick leave, adequate housing, and universal healthcare access [49]. Syndemic approaches necessitate integrated programs addressing co‐occurring conditions, for example, combining HIV prevention with poverty alleviation, mental health support, and food security interventions [23].

#### 3.4.1 Critical Synthesis

Digital epidemiology raises important ethical concerns regarding privacy, data ownership, and algorithmic bias that require urgent attention [50]. The commercial availability of mobility data, primarily from smartphone users, may systematically exclude populations without digital access, potentially perpetuating rather than reducing health disparities. Agent‐based models, while powerful, require extensive parameterization and validation; their complexity can obscure rather than illuminate mechanisms. Evaluations of stigma reduction and communication interventions show mixed effectiveness, with many studies lacking rigorous designs. The evidence base for structural interventions remains limited by the difficulty of conducting randomized trials of policy changes.

## 4 Discussion

The evidence synthesized in this review demonstrates that infectious diseases cannot be understood through purely biomedical lenses; they are fundamentally biosocial phenomena requiring integrated analytical frameworks. The main contribution of this review is the integrative synthesis of syndemic and social contagion theories to demonstrate how social processes operate across multiple levels to affect disease transmission, rather than the introduction of a wholly novel theoretical framework. The study's findings need assessment because both evidence limitations and review restrictions need consideration before analyzing their impact.

The literature on social dimensions of infectious diseases, while extensive, exhibits important weaknesses. First, study design limitations restrict causal inference because most studies use cross‐sectional and ecological designs, which create confounding and reverse causality problems for social factors and disease outcomes. Second, measurement heterogeneity across studies, with different operationalizations of socioeconomic status, social capital, and trust, complicates synthesis and limits generalizability. Third, publication bias may lead to overrepresentation of positive findings, particularly for novel interventions. Fourth, high‐income countries receive primary research attention, which results in frameworks that developed in these countries to become irrelevant for low‐ and middle‐income areas because their social systems and health systems, and disease patterns differ from those in high‐income countries. Fifth, the rapid evolution of digital technologies outpaces evaluation, with policy decisions often preceding evidence.

**Figure 1:**
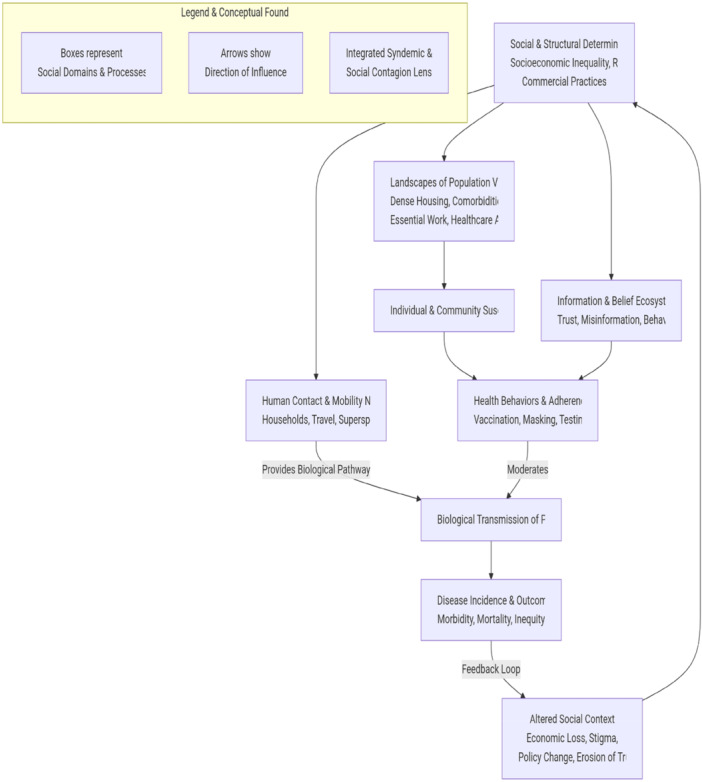
Integrated model of social contagion in infectious disease dynamics. Four interconnected domains: Structural foundations (macro‐level: inequality, policy) shape network infrastructure (meso‐level: contact patterns, mobility), which influences Information‐behavior dynamics (micro‐level: trust, behavioral contagion) and ultimately pathogen transmission (biological level). Feedback loops: disease outcomes reshape structural conditions, modify information environments, and alter contact patterns. Intervention points operate at all levels: structural policies, network‐targeted restrictions, information strategies, and socially‐contextualized biomedical tools.

The syndemic framework, originating in medical anthropology, emphasizes how adverse social conditions create synergistic interactions between diseases [3, 23]. Social contagion theory, originating in sociology and network science, explains how behaviors, ideas, and affect spread through social networks [2, 10]. The integrative synthesis presented here reveals that syndemic conditions themselves spread through social contagion processes: vulnerability factors cluster not only ecologically but through network mechanisms, as disadvantage propagates through social ties. Conversely, social contagion of protective behaviors, vaccination, and health‐seeking can mitigate syndemic vulnerability. This integrated lens suggests that interventions targeting network hubs for information dissemination may simultaneously address multiple syndemic components, as illustrated in Figure 1.

The first thematic area of this review, structural determinants, and the second thematic area of network effects, and the third thematic area of belief systems, and the fourth thematic area of digital tools create an interconnected system that operates through their combined effects according to the diagram in Figure 1. Structural conditions create contact networks through residential segregation by income, which produces homogeneous contact networks, and commercial marketing creates information environments that specifically target disadvantaged communities. Network structures determine how pathogens and information propagate. The structural position and network exposure of a person shape their beliefs, which direct their conduct that affects disease transmission. Digital tools both reveal and reshape these dynamics, which generate feedback loops that drive social contagion processes. The systems perspective demands that effective interventions should work at multiple levels.

A significant limitation acknowledged throughout this review is that research in high‐income countries makes up most of this review, which reflects existing literature, yet does not allow researchers to determine its worldwide applicability. Social structures in low‐ and middle‐income countries differ in ways that likely modify social contagion dynamics: multigenerational households are more common, informal settlements create high‐density networks, collectivist cultural orientations may shape behavioral contagion differently, and health systems face resource constraints that alter intervention possibilities [51]. The rapid urbanization occurring across Africa and Asia creates novel contact patterns whose implications for disease transmission remain understudied. Future research must prioritize diverse geographic settings and develop culturally grounded measures of social processes.

Methodological and modeling implications: The process of integrating social factors into mathematical models that study infectious disease spread remains difficult, yet it serves as an essential requirement. Current approaches include: (1) stratifying compartmental models by socioeconomic or occupational groups; (2) incorporating empirically‐derived contact matrices that capture age‐specific and setting‐specific mixing; (3) agent‐based models that simulate heterogeneous populations with social network structures; and (4) metapopulation models that represent geographic movement patterns. Each approach involves trade‐offs between mechanistic detail, parameter identifiability, and computational tractability. Future advances require closer collaboration between modelers, social scientists, and empirical researchers to ensure that models capture socially meaningful heterogeneity while remaining grounded in observable data.

Policy and practice implications: The biosocial perspective that this research presents to the public contains specific effects that influence both policy development and practical implementation. Surveillance systems should monitor not only pathogen distribution but also social vulnerability indicators, misinformation trends, and trust levels. Preparedness planning must address structural determinants through cross‐sectoral collaboration with housing, labor, and education sectors. The design of response strategies should involve affected communities because trust develops through respectful treatment, which builds true partnerships. Equity must function as the primary outcome measurement, which requires assessment of how interventions affect distributional outcomes across various social groups.

As a narrative review, this synthesis has several limitations. The selection of literature, while systematic in search strategy, involved subjective judgments about relevance and importance. The absence of formal quality appraisal means that study limitations are discussed qualitatively rather than systematically assessed. The broad interdisciplinary scope inevitably sacrifices depth in any single field for conceptual integration, and readers seeking detailed treatment of specific topics should consult specialized reviews. The review period (2000–2024) captures the digital transformation of social life, but may miss earlier foundational work not fully represented. Finally, the author's positionality as a researcher trained in biomedical and public health traditions shapes the interpretation presented. For future researchers, Longitudinal studies are needed to establish causal pathways linking structural factors to infection risk, incorporating dynamic network data to improve epidemic models. Rigorous evaluations of misinformation interventions and structural policies (housing, labor, anti‐discrimination laws) are essential, with research prioritized in low‐ and middle‐income countries. Ultimately, recognizing pandemics as reflections of underlying social patterns is critical for developing effective prevention and response strategies.

## 5 Conclusions

The “whispers” of pathogens, their transmission, amplification, and eventual control are fundamentally shaped by the social world. This review has articulated how structural determinants create landscapes of vulnerability, how human networks define transmission pathways, how information ecosystems shape protective behaviors, and how digital tools both reveal and reshape these dynamics. The social determinants of health are, inescapably, determinants of infectious disease dynamics.

### 5.1 Key Conclusions

1. People require social epidemiology as the basic framework for studying infectious disease epidemiology. The three elements of socioeconomic factors, demographic details, and behavioral patterns must be treated as essential elements of surveillance, modeling, and intervention design processes.
2. Equity serves as the essential element that enables successful epidemic readiness and response activities. The three policies that address structural inequalities through living wages, proper housing, and complete healthcare access function as broad‐spectrum antimicrobials that decrease vulnerability to multiple pathogens.
3. Public health depends on trust as a crucial social asset. The process of building trust needs people to establish transparent systems that work together with the community while taking responsibility for past actions to show that all decisions will prioritize population health above political and commercial purposes.
4. Syndemic approaches are required to treat multiple diseases, which together create complex health challenges. Integrated interventions that tackle common social determinants are necessary when disadvantaged communities experience outbreaks of infections, together with non‐communicable diseases and mental health disorders.
5. Digital transformation has brought complete changes to how social contagion functions by providing organizations with new chances through real‐time surveillance and targeted communication, while facing obstacles from misinformation spreading and privacy issues which create needs for continuous adaptation.

## Author Contributions

**Sayed Mortaza Fayez:** investigation, conceptualization, writing – review and editing, methodology, formal analysis, writing – original draft, and data curation.

## Funding

The author has nothing to report.

## Conflicts of Interest

The author declares no conflicts of interest.

## Transparency Statement

The lead author, Sayed Mortaza Fayez, affirms that this manuscript is an honest, accurate, and transparent account of the study being reported; that no important aspects of the study have been omitted; and that any discrepancies from the study as planned (and, if relevant, registered) have been explained.

## Data Availability

Data sharing is not applicable to this article as no new data were created or analyzed in this study. All data underlying the findings of this narrative review are derived from published sources cited in the references.
